# A Siamese ResNeXt network for predicting carotid intimal thickness of patients with T2DM from fundus images

**DOI:** 10.3389/fendo.2024.1364519

**Published:** 2024-03-14

**Authors:** AJuan Gong, Wanjin Fu, Heng Li, Na Guo, Tianrong Pan

**Affiliations:** ^1^ Department of Endocrinology, The Second Affiliated Hospital of Anhui Medical University, Hefei, China; ^2^ Department of Clinical Pharmacology, The Second Affiliated Hospital of Anhui Medical University, Hefei, China; ^3^ The Department of Computer Science and Engineering, Southern University of Science and Technology, Shenzhen, China; ^4^ School of Computer and Communication Engineering, University of Science and Technology Beijing, Beijing, China

**Keywords:** carotid intima-media thickness, type 2 diabetes mellitus, deep neural networks, retinal fundus images, ResNeXt

## Abstract

**Objective:**

To develop and validate an artificial intelligence diagnostic model based on fundus images for predicting Carotid Intima-Media Thickness (CIMT) in individuals with Type 2 Diabetes Mellitus (T2DM).

**Methods:**

In total, 1236 patients with T2DM who had both retinal fundus images and CIMT ultrasound records within a single hospital stay were enrolled. Data were divided into normal and thickened groups and sent to eight deep learning models: convolutional neural networks of the eight models were all based on ResNet or ResNeXt. Their encoder and decoder modes are different, including the standard mode, the Parallel learning mode, and the Siamese mode. Except for the six unimodal networks, two multimodal networks based on ResNeXt under the Parallel learning mode or the Siamese mode were embedded with ages. Performance of eight models were compared via the confusion matrix, precision, recall, specificity, F1 value, and ROC curve, and recall was regarded as the main indicator. Besides, Grad-CAM was used to visualize the decisions made by Siamese ResNeXt network, which is the best performance.

**Results:**

Performance of various models demonstrated the following points: 1) the RexNeXt showed a notable improvement over the ResNet; 2) the structural Siamese networks, which extracted features parallelly and independently, exhibited slight performance enhancements compared to the traditional networks. Notably, the Siamese networks resulted in significant improvements; 3) the performance of classification declined if the age factor was embedded in the network. Taken together, the Siamese ResNeXt unimodal model performed best for its superior efficacy and robustness. This model achieved a recall rate of 88.0% and an AUC value of 90.88% in the validation subset. Additionally, heatmaps calculated by the Grad-CAM algorithm presented concentrated and orderly mappings around the optic disc vascular area in normal CIMT groups and dispersed, irregular patterns in thickened CIMT groups.

**Conclusion:**

We provided a Siamese ResNeXt neural network for predicting the carotid intimal thickness of patients with T2DM from fundus images and confirmed the correlation between fundus microvascular lesions and CIMT.

## 1 Introduction

Over 500 million patients with Type 2 Diabetes Mellitus (T2DM) (1, 2) globally are taking the high risk of macrovascular complications like cardiac, cerebral, and peripheral vessels (3). These complications may significantly increase the probability of morbidity and mortality. Carotid Intima-Media Thickness (CIMT) is a pivotal biomarker for assessing macrovascular pathologies (4, 5). In patients with diabetes, thickened CIMT signals the early onset of atherosclerosis, thereby elevating the risk for cardiovascular incidents, including heart disease and stroke (6). Early detection of CIMT of course makes sense to T2DM patients. However, conventional checking methods like CT imaging evaluation and carotid artery ultrasound examination are expensive and cannot be performed routinely, especially in developing or underdeveloped regions. Many T2DM patients cannot receive early therapeutic intervention (7).

Fundus imaging is universally known as an indispensable routine screening modality for T2DM (8). Biologically, the ophthalmic artery is an integral subsidiary of the internal carotid artery and the leading vascular provider for the retina (9); variation of the hemodynamics of the internal carotid artery definitely may result in anomalies of the retinal microvasculature (9). Fundus images can be an indirect barometer of systemic disease (10–12). More notably, artificial intelligence (AI) predicting methods from retinal images, which have significant advantages in multifactorial issues with high-dimensional data, has been widely applied in systemic disease diagnostics, such as cardiovascular diseases (13), cerebrovascular accidents (14), chronic renal disorders Alzheimer’s disease (15), and carotid artery stenosis (16).

Based on the fact that changes in retinal microvasculature can reflect internal carotid artery (17–19), Junlong Qu (16) proposed a multimodal fusion predicting model based on fundus images and clinical indices, which can detect carotid artery stenosis automatically. Although the model’s accuracy (74.82%) is not high enough, the research confirmed that predicting CIMT for patients with T2DM from fundus images using deep neural networks is a potential method (16).

For early detection of CIMT in T2DM, which can consequently benefit patients from the prevention of cardiovascular diseases via early intervention, this paper established a specific fundus images dataset and proposed a Siamese ResNeXt network for predicting CIMT. The accuracy of Siamese ResNeXt is 88.0%, which further confirms the correlation between CIMT and retinal abnormalities and provides a valuable tool for early detection of CIMT in patients with T2DM.

## 2 Materials and methods

### 2.1 Dataset

#### 2.1.1 Diagnostic criteria

##### 2.1.1.1 The criteria for the diagnosis of T2DM: 

Patients have diabetes-specific symptoms, like xerostomia, polydipsia, polyuria, and inexplicable weight reduction, and the random plasma glucose level of who is equal to or exceeding 11.1 mmol/L; the fasting plasma glucose level after necessitating a minimum fast of eight hours equal to or exceeding 7.0 mmol/L; the postprandial plasma glucose level two hours after 75g oral glucose is equal to or exceeding 11.1 mmol/L (20).

##### 2.1.1.2 The diagnostic benchmarks for CIMT:

The CIMT has not yet been clear in clinical, due to differences in ethnicity, age, and measuring equipment. Luca SABA and others (21) drew that the CIMT thickness threshold were between 0.7 millimeters and 1.2 millimeters, from 107 global studies on the correlation between CIMT thickness and vascular diseases. In this paper, based on the research of Chinese population (22), patients are classified into the normal group if their CIMT is less than 0.9 mm and the thickened group if their CIMT is over or equal to 0.9 mm

#### 2.1.2 Inclusion and exclusion criteria

##### 2.1.2.1 Inclusion criteria

(1) Individuals must be 18 years or older, with no restrictions based on gender.
(2) Participants must meet the diagnostic benchmarks for T2DM as established guidelines stipulate.
(3) The participants must have complete clinical records readily available for research evaluation.

##### 2.1.2.2 Exclusion criteria

(1) Patients diagnosed with type 1 diabetes mellitus, gestational diabetes, or other specific diabetes variants are precluded from the study.
(2) Patients with archived ophthalmic images of suboptimal quality, which precludes the extraction of valuable data for the study, are excluded.
(3) Patients whose archived ultrasonographic assessments of the carotid arteries fail to detail the measurements of the CIMT are also excluded from participation.

#### 2.1.3 Data collection

**Figure 1:**
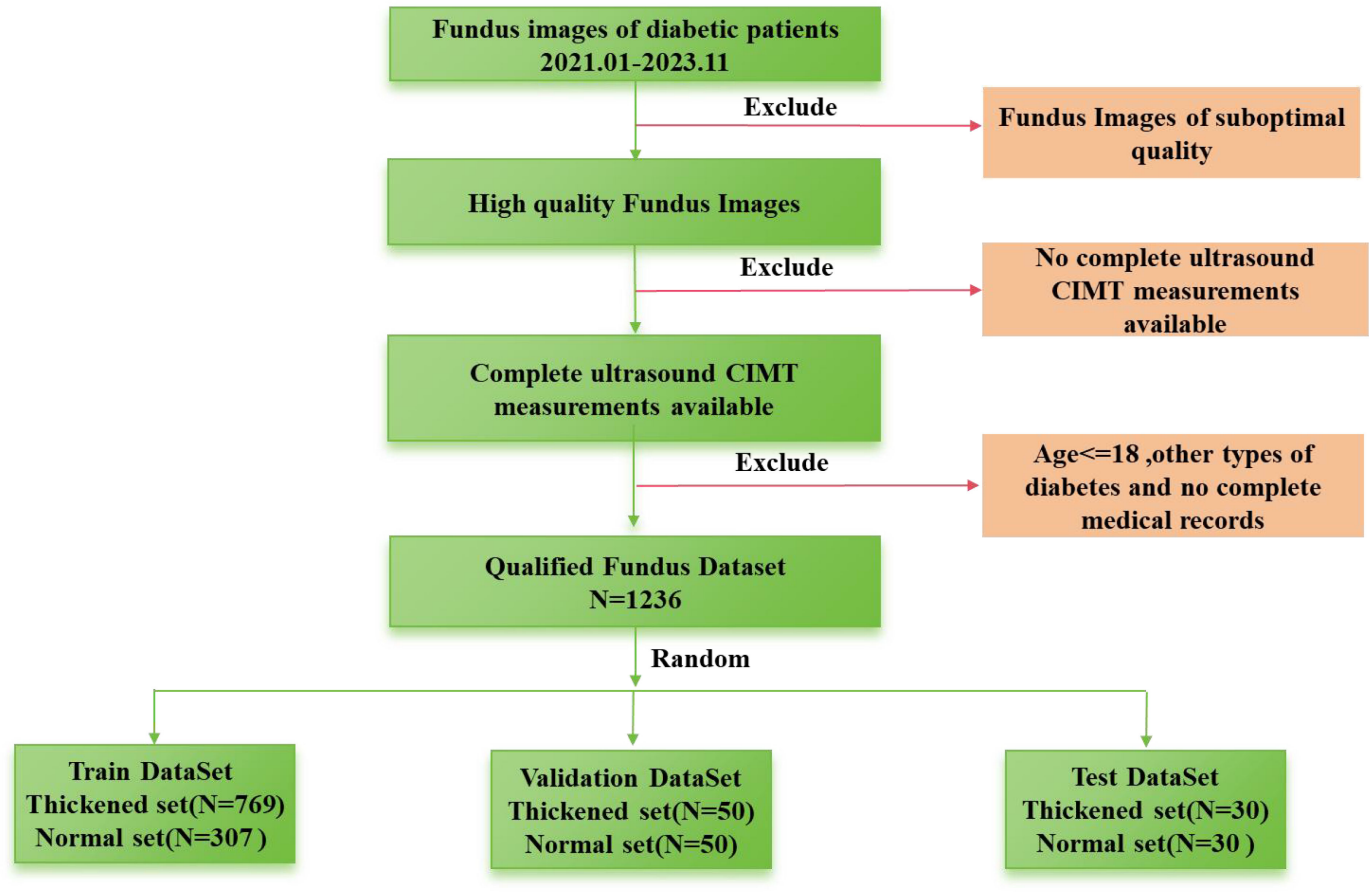
Flow chart of data collection.

This retrospective case-control investigation systematically assessed a cohort of individuals diagnosed with T2DM hospitalized in the Second Affiliated Hospital of Anhui Medical University from January, 2021 to November, 2023. After excluding subjects with non-qualifying ophthalmic fundus images, the study encompassed a sample size of, 1236 patients. The dataset is randomly divided into test, validation, and training groups. The validation group consists of 50 normal patients and 50 patients with thickening, while the test group includes 30 normal patients and 30 patients with thickening. The remaining data are allocated to the training group. The process of dataset collection is illustrated in **Figure 1**. Their clinical parameters (including sex, age, and hospital admission identifiers), high-resolution fundus photographs, and CIMT values determined by ultrasonography were acquired. This research has received formal approval from the Ethics Committee of the aforementioned hospital. The approval number is YX2023-2011(F1).

**Figure 2:**
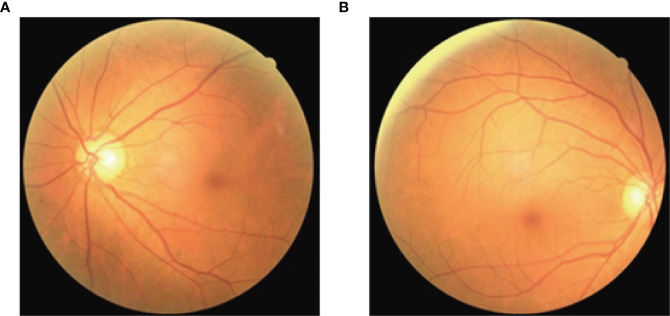
Fundus images. **(A)**. Left fundus image; **(B)**. Right fundus image.

**Figure 3:**
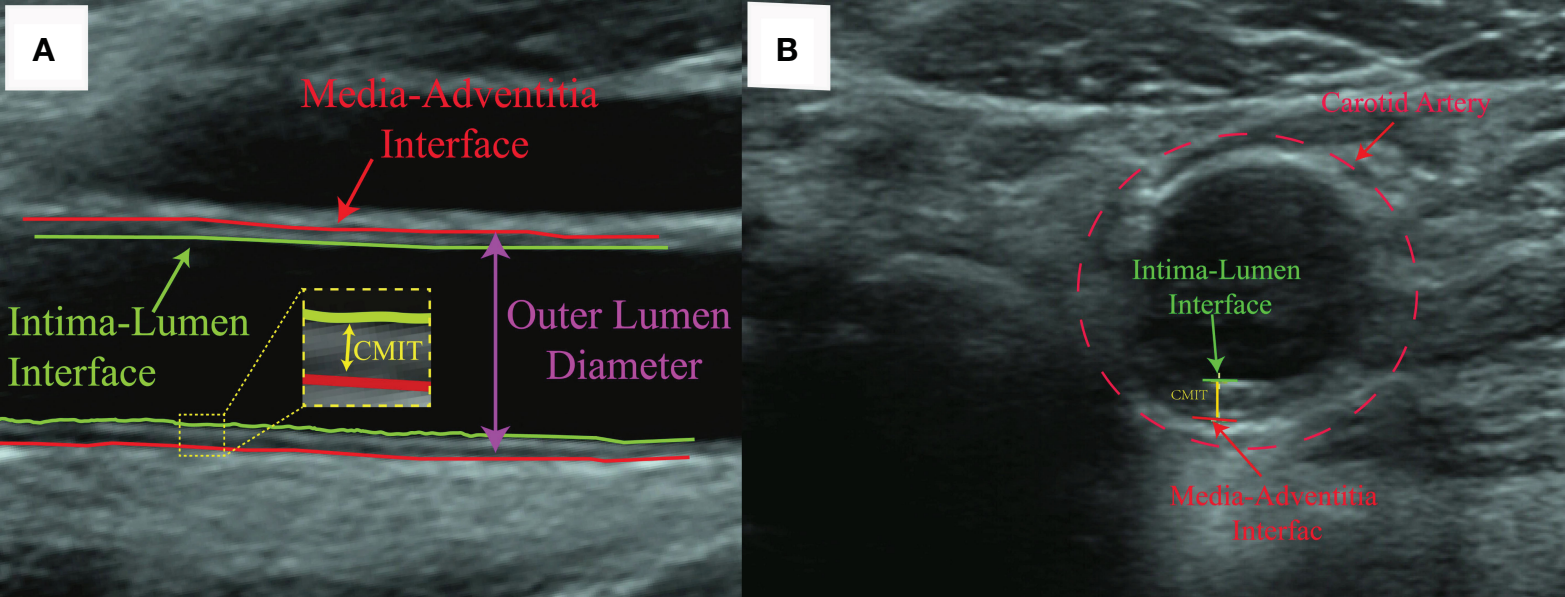
Ultrasound images of the cross-sectional and longitudinal sections for measuring the thickness of CMIT. **(A)** is a longitudinal ultrasound view of the carotid artery, where the green line indicates the Intima-Lumen Interface and the red line marks the Media-Adventitia Interface, with the CIMT measured in the yellow dashed area. **(B)** is a cross-sectional ultrasound view of the carotid artery, with the CIMT located between the green and red dashed lines, enclosed by the red dashed circle indicating the artery’s boundary.

Fundus imagery was obtained utilizing the Canon CR-2 PLUS AF non-mydriatic digital fundus photography apparatus, which facilitated the capture of images depicting the natural dilation of the pupils at a 45-degree acquisition angle without the necessity of pharmacologic pupillary dilation, show in **Figure 2**. The measurement of CIMT was conducted by professional sonographers in the hospital’s ultrasound department using a Siemens ACUSON S2000 ultrasound diagnostic instrument, which is equipped with an L16 transducer with a frequency range of 5-12 MHz, while the patient was at rest with their head turned to the side. The detailed methodology for measuring the CIMT is as follows: Initially, the precise location for the measurement must be identified, typically targeting the far wall of the common carotid artery (CCA), specifically about 1-2 cm above the carotid bulb. This area is chosen because of its relatively flat surface and the distinct clarity of the interface between the intima and media layers, which enables the capture of high-quality images. Subsequent steps involve pinpointing the carotid artery and acquiring both transverse and longitudinal sectional images to accurately determine the measurement point. The actual CIMT measurement is conducted on the longitudinal section, calculating the distance between the intima-lumen interface and the intima-media interface, show in **Figure 3**.

#### 2.1.4 Privacy protection

In the initial stages of data collection, rigorous measures were implemented to safeguard patient privacy rights comprehensively. This involved anonymizing all clinical data that could contain identifiable markers, thereby securing the confidentiality of personal information. Furthermore, fundus images were meticulously cropped to excise any segments potentially comprising individual identification elements. Throughout the entirety of the data collection and processing trajectory, the study carefully conformed to predefined standard operating procedures, guaranteeing data uniformity and comparability and thereby maintaining the scientific rigor and ethical integrity of the research endeavor.

### 2.2 data processing

In pursuit of a robust generalization for the model, a refined palette of geometric and photometric augmentation techniques was integrated to expand the dataset. Geometric augmentations are articulated through a stochastic rotation algorithm that adjusts random orientation within a precisely controlled angular spectrum of ±5 degrees, meticulously retaining the image’s central fidelity. A bidirectional flipping in horizontal and vertical was implemented to enrich the model’s interpretative versatility across variously oriented planes. Photometric augmentations, which delicately fine-tune the imagery’s luminosity, contrast, saturation, and hue, were executed randomly after geometric enlargements, presenting diverse visual scenarios. Such deliberate and strategic data augmentation strategy primed the model for consistent and reliable performance under different imaging environments, thereby solidifying its practicality and robustness in real-world clinical applications.

### 2.3 Diagnostic model

#### 2.3.1 Network architecture

Typically, there are two types of AI diagnostics models for systemic disease. The feature-driven analytical models depended on a definite correlation between image characters and disease. The VGGNet-16 network for assessing the risk of ischemic stroke (23) is based on the correlation between vascular caliber and cerebrovascular events (24). However, other excluded features may be ignored. Although the feature-free model is weakly interpretable, it is efficient, especially if classification characteristics are unclear. Even though the details of retinal vascular were enhanced, their features were not given in the predicting model of biological age based on the VGG-19 network (25).

In spite of the significant correlation between CIMT and retinal abnormalities (19), biological characteristics are not pointed. Accordingly, feature-free deep learning algorithms based on two popular deep learning frameworks, ResNet (26) and ResNeXt (27), were used to predict CIMT.

**Figure 4:**
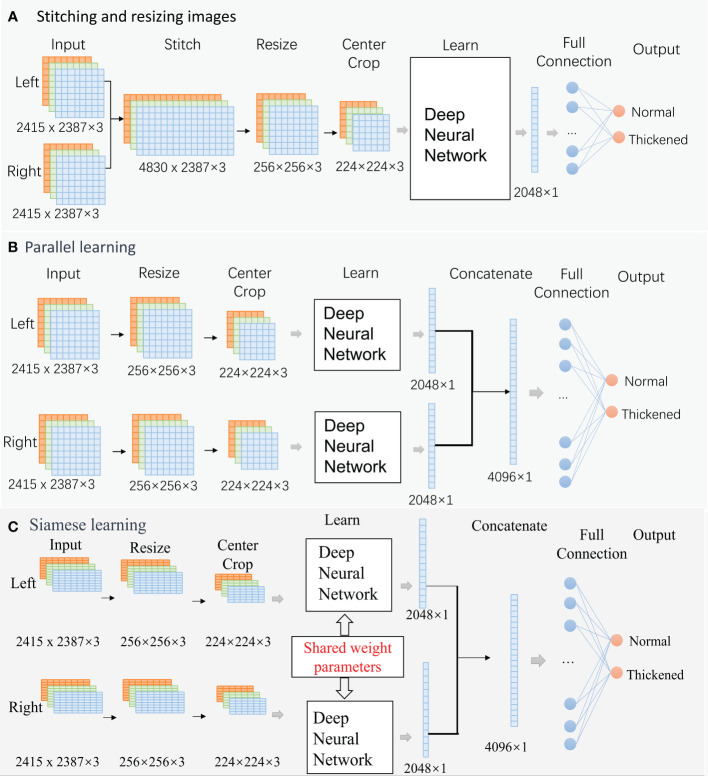
Classification Networks. **(A)**. Stitching and resizing images; **(B)**. Parallel learning; **(C)**. Siamese learning.

The encoder and decoder can affect the classification results. Considering that a group of fundus images comprises images from the left and right eyes, pairs of images should be trained simultaneously. Three different encoder and decoder modes were designed in this paper (**Figure 4**). Besides, based on the correlation between age and CIMT thickening in demographic statistics, multimodal modes with the aged were also trained. Therefore, eight prediction models were trained in experiments.

The raw images are RGB images with a resolution of, 2415 × 2387. The deep neural algorithms in each mode are ResNet50 or ResNeXt50 in this paper.

##### 2.3.1.1 Mode A: Stitching and resizing images

Two raw images were stitched into one image (4830× 2387×3). Then, the stitched image was resized to 256×256×3. A center cropping operation is performed to obtain an image area of 224×224×3 before learning. An output feature vector of, 2048×1 was extracted from the deep neural network. The vector is fed into a fully connected layer for classification.

##### 2.3.1.2 Mode B: Parallel learning

This is a structural parallel network. The architecture of the two networks is the same, but their parameters can be different. Firstly, two raw images (2415 × 2387×3) were resized to 256×256×3, and center cropped to 224×224×3 independently. Then, the processed images were fed into two deep neural networks independently for learning. The networks’ two output feature vectors, 2048×1, were concatenated into a large vector of, 4096×1, and the large vector was fed into a fully connected layer for classification.

##### 2.3.1.3 Mode C: Siamese learning

This is a siamese network, and the architecture and parameters value of the two sub-networks is identical. These networks follow the same path as ModeB during forward propagation.

##### 2.3.1.4 Multimodal mode

The primary frameworks of the multimodal mode are similar to that of the unimodal mode. The significant difference is that the 128-dimensional age vectors and the output feature vector of deep neural networks were concatenated into one vector before being fed into a fully connected layer for classification.

#### 2.3.2 Model optimization and loss functionality

A gradient-based optimization called Adam was adopted in this paper. The Adam optimizer can calculate the adaptive weighted moving averages of both gradients and their squared values,updates requisite for model training and convergence with pronounced efficiency (28). The formula for calculating the Adam optimizer can be found in Equation 1 (28).

In light of the pronounced imbalance in sample sizes among different categories within our research dataset, we have adopted a weighted cross-entropy loss function for this classification task. This loss function can assign weights to the numbers of each class. Categories with fewer samples are allocated higher weights. Such a method can ensure that all categories can be classified accurately, particularly for those small categories.The calculation of these weights is specified in Equation 2.


(1) $$H\left(p,q\right)=\;-\sum_{i=1}^{N}w_{i}p\left(x_{i}\right)\log\left(q\left(x_{i}\right)\right)$$

Within the equation, p(xi​) epitomizes the ground truth associated with the i-th label, while q(xi​) corresponds to the estimated predictive value, N denotes the whole number of data; n1 denotes the number of data of normal groups; n2 denotes the number of data of thickened groups.


(2) $$w_{i}=\{\begin{array}{c} \frac{n_{1}}{N},\;\;\;\;if\;x_{i}\in \left\{\text{normal}\;\text{groups}\right\}\;\\ \frac{n_{2}}{N},\;\;if\;x_{i}\in \left\{\text{thickened}\;\text{groups}\right\} \end{array}$$

#### 2.3.3 Assessment indicators

**Common indicators**:Confusion matrix, precision, recall, specificity, F1 Score and ROC curve are used to evaluate the performance of different deep neural networks. The formulas for calculating the metrics precision, recall, specificity, and F1 Score can be found in Equations 3–9.


(3) $$\text{Precision}\_\text{Thickened}=\frac{\text{Num}\_\text{TT}}{\text{Num}\_\text{TT}+\text{Num}\_\text{TN}}$$

(4) $$\text{Recall}\_\text{Thickened}=\frac{\text{Num}\_\text{TT}}{\text{Num}\_\text{TT}+\text{Num}\_\text{NN}}$$

(5) $$\text{Specificity}\_\text{Thickened}=\frac{\text{Num}\_\text{NT}}{\text{Num}\_\text{NT}+\text{Num}\_\text{TN}}$$

(6) $$\text{Precision}\_\text{Normal}=\frac{\text{Num}\_\text{NT}}{\text{Num}\_\text{NT}+\text{Num}\_\text{NN}}$$

(7) $$\text{Recall}\_\text{Normal}=\frac{\text{Num}\_\text{NT}}{\text{Num}\_\text{NT}+\text{Num}\_\text{TN}}$$

(8) $$\text{Specificity}\_\text{Normal}=\frac{\text{Num}\_\text{TT}}{\text{Num}\_\text{TT}+\text{Num}\_\text{NN}}$$

(9) $$\text{F}1\text{ Score}=2*\frac{\text{Precision}*\text{Recall}}{\text{Precision}+\text{Recall}}$$

Note: Num_TT: The number of thickened group instances correctly identified as belonging to the thickened group. Num_TN: The number of normal group instances incorrectly identified as belonging to the thickened group. Num_NT: The number of thickened group instances incorrectly identified as belonging to the normal group. Num_NN: The number of normal group instances correctly identified as belonging to the normal group.Precision: The ratio of correctly predicted positive observations to the total predicted positives.Recall : The ratio of correctly predicted positive observations to all observations in actual class.Specificity: The measure of the ability of the model to correctly identify negatives.F1 Score: The weighted average of Precision and Recall. Therefore, this score takes both false positives and false negatives into account.

##### 2.3.3.1 Clinical indicators:

Specifically noted that, in clinical application, other than adding extra checks, the misdiagnosis that normal patients are classified into the thickening group of the carotid artery intima cannot produce serious consequences. However, the missed diagnosis that patients with thickening of the carotid artery intima have not been screened may delay early intervention for patients. Therefore, the recall of the thickened group is equally important as the overall accuracy of the classification model. The recall of the normal group is inferior to that of the thickened group and the overall model.

#### 2.3.4 Class activation map

Activation Maximization (AM) (29), Deconvolutional Neural Network Visualization (DeconvNet) (30), Class Activation Mapping (31), and other methods are employed to enhance the transparency and interpretability of the black-box prediction model.

This paper overlaps the heatmaps calculated through the Grad-CAM technique onto the input image to highlight the areas that contributed the most to the network’s decision. First, the gradients of the predicted class score concerning the final convolutional layer’s feature map, which were obtained when the input image was passed through the classification network, are computed. Then, the average gradient value for each channel is calculated using global average pooling. Finally, the feature maps were linearly weighted by their corresponding gradient values, then ReLU-activated and aligned to the original input image size (32, 33).

### 2.4 Training

All eight models were trained under the same training strategy, with the entire process spanning 400 epochs. The process is divided into two main phases: In the foundational training phase, models initially load parameters pre-trained on ImageNet and are trained using the Adam optimizer. The initial learning rate is set to 0.001, with a decay to 10% of its value every 10 epochs. The batch size is set to 32 for all model types, including the Parallel, Standard, and Siamese models. The best-performing model parameters on the validation set are saved during this phase. In the subsequent 300 epochs of advanced training, models load the best-performing parameters from the foundational training and adjust the learning rate to either 0.0001 or 0.00005, with all other hyperparameters remaining unchanged, to conduct in-depth advanced training. After completing this series of training, the models’ capabilities are comprehensively evaluated on the test set.

Besides, the Grad-CAM was used to help highlight which regions of an input image after the prediction model was trained. This paper calculated the heatmaps only for the Siamese ResNeXt model, the performance of which was best.

## 3 Results

### 3.1 Demographic information

In this retrospective analysis, the dataset encompassed 1,236 subjects, categorized into the CIMT-normal group with 387 individuals (31.31%) and the CIMT-thickened group with 849 individuals (68.69%). Subsequent subgroup analysis delineated a mean age of 37.33 ± 9.95 years for the CIMT-normal cohort. Conversely, the CIMT-thickened group exhibited an elevated mean age of 53.74 ± 9.99 years. Statistical evaluation revealed a statistically significant divergence in age distribution between the CIMT-normal and thickened cohorts (P< 0.001), indicating a pronounced correlation between age and the variation in CIMT measurements, shown in **Table 1**.

**Table 1:** Demographic Characteristics by CIMT Category.

	Normal Group			Thickened Group		
	Male N=280	Female N=107	Total N=387	Male N=531	Female N=318	Total N=849
**Age**	35.90 ± 8.58	41.08 ± 12.13	37.33 ± 9.95	51.41 ± 10.41^***^	57.64 ± 7.84^***^	53.74 ± 9.99^***^

***, p<0.001.

### 3.2 Performance of prediction models

**Figure 5:**
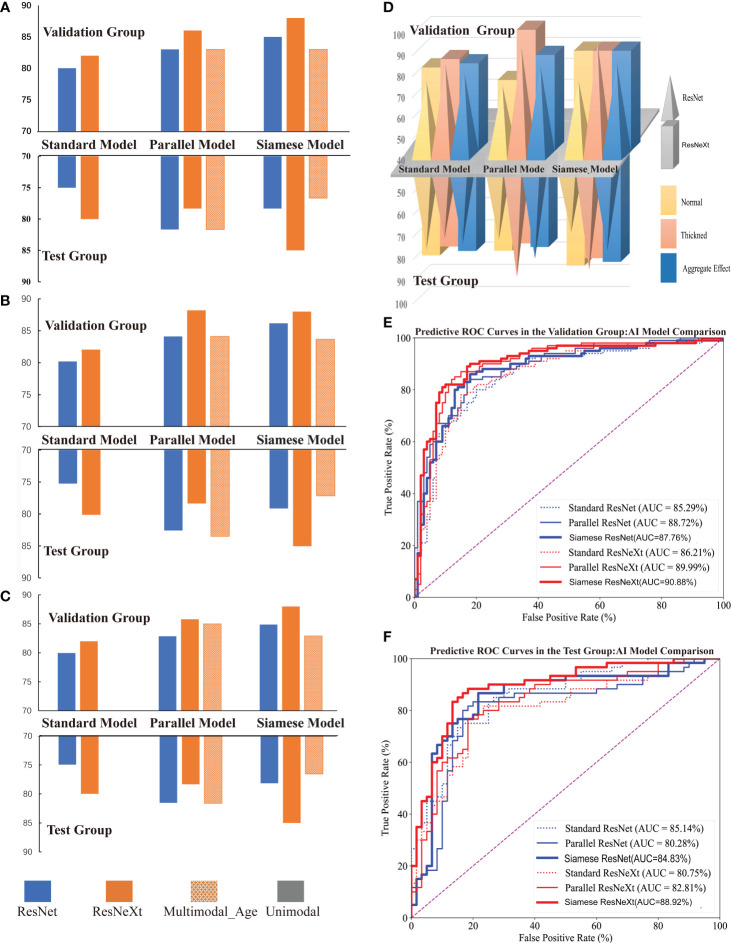
Performance of Different Models for Predicting CIMT Thickness. **(A–C)** illustrate the comparative performance metrics of recall rate, precision, and F1 score for various CIMT prediction models. **(D)** displays the performance of models in terms of recall rate across average, thickened, and aggregate effects for predicting CIMT. **(E, F)** show the ROC curves and AUC of various deep learning models in the test and validation groups for CIMT prediction.

**Figure 6:**
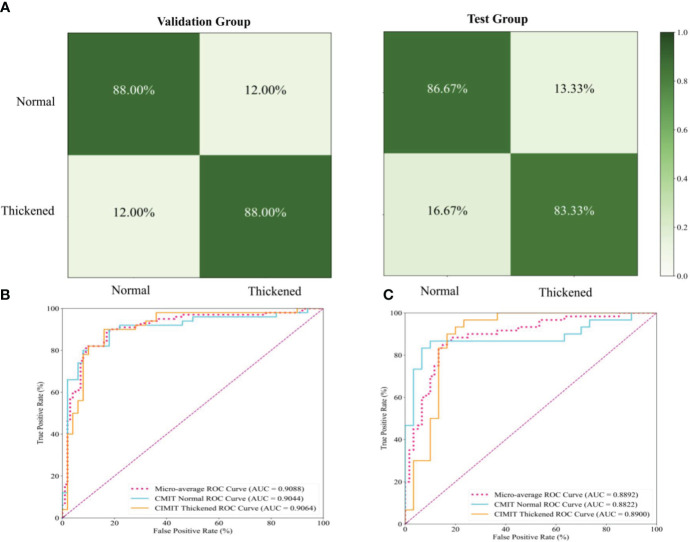
Performance Evaluation of the Siamese ResNeXt Model Using Fundus Images for the Prediction of Carotid Artery Thickness. **(A)** displays the confusion matrices for the Siamese ResNeXt model’s prediction of CIMT in both validation and test groups. **(B, C)** show the ROC curves and AUC performance of the Siamese ResNeXt model in the valid and test groups.

The names of various predictive models are presented in **Table 2**. The performance of these models is illustrated in **Table 3**, **Figures 5**, **6**.

**Table 2:** Names of different predicting models.

Model Name	Encoder and decoder mode	Deep Neural Network	Data Modality
Standard ResNet	Stitching and resizing images	ResNet	unimodal modes
Parallel ResNet	Parallel learning	ResNet	unimodal modes
Siamese ResNet	Siamese learning	ResNet	unimodal modes
Standard ResNext	Stitching and resizing images	ResNeXt	unimodal modes
Parallel ResNeXt	Parallel learning	ResNeXt	unimodal modes
Siamese ResNeXt	Siamese learning	ResNeXt	unimodal modes
Parallel ResNeXt & Age	Parallel learning	ResNeXt	multimodal mode
Siamese ResNext & Age	Siamese learning	ResNeXt	multimodal mode

The prediction model, which is stitching and resizing raw images, is a standard ResNet/ResNeXt classification model.

**Table 3:** Performance of different predicting models.

Model Name	Data Group	Validation Group Results					Test Group Results				
		Precision	Recall	Specificity	F1-score	AUC	Precision	Recall	Specificity	F1-score	AUC
**Standard ResNet**	**Normal**	82.61%	76.00%	84.00%	79.17%	85.00%	77.78%	70.00%	80.00%	73.68%	86.67%
	**Thickened**	77.78%	84.00%	76.00%	80.77%	84.29%	72.73%	80.00%	70.00%	76.19%	85.44%
	**Average**	80.19%	80.00%	80.00%	79.97%	85.29%	75.25%	75.00%	75.00%	74.94%	85.14%
**Parallel ResNet**	**Normal**	90.24%	74.00%	92.00%	81.32%	89.68%	88.00%	73.33%	90.00%	80.00%	79.89%
	**Thickened**	77.97%	92.00%	74.00%	84.40%	89.84%	77.14%	90.00%	73.33%	83.08%	79.89%
	**Average**	84.11%	83.00%	83.00%	82.86%	88.72%	82.57%	81.67%	81.67%	81.54%	80.28%
**Siamese ResNet**	**Normal**	92.68%	76.00%	94.00%	83.52%	88.12%	84.00%	70.00%	86.67%	76.36%	85.22%
	**Thickened**	79.66%	94.00%	76.00%	86.24%	88.76%	74.29%	86.67%	70.00%	80.00%	84.67%
	**Average**	86.17%	85.00%	85.00%	84.88%	88.76%	79.14%	78.33%	78.33%	78.16%	84.83%
**Standard ResNeXt**	**Normal**	83.33%	80.00%	84.00%	81.63%	86.20%	82.14%	76.67%	83.33%	79.31%	77.20%
	**Thickened**	80.77%	84.00%	80.00%	82.35%	86.96%	78.12%	83.33%	76.67%	80.65%	84.11%
	**Average**	82.05%	82.00%	82.00%	81.99%	86.21%	80.13%	80.00%	80.00%	79.98%	80.75%
**Parallel ResNeXt**	**Normal**	97.37%	74.00%	98.00%	84.09%	90.64%	77.42%	80.00%	76.67%	78.69%	82.44%
	**Thickened**	79.03%	98.00%	74.00%	87.50%	89.04%	79.31%	76.67%	80.00%	77.97%	83.67%
	**Average**	88.20%	86.00%	86.00%	85.80%	89.99%	78.36%	78.33%	78.33%	78.33%	82.81%
**Siamese ResNeXt**	**Normal**	88.00%	88.00%	88.00%	88.00%	90.44%	83.87%	86.67%	83.33%	85.25%	88.22%
	**Thickened**	88.00%	88.00%	88.00%	88.00%	90.64%	86.21%	83.33%	86.67%	84.75%	89.00%
	**Average**	88.00%	88.00%	88.00%	88.00%	90.88%	85.04%	85.00%	85.00%	85.00%	88.92%
**Parallel ResNeXt & Age**	**Normal**	90.24%	74.00%	92.00%	81.32%	88.36%	91.30%	70.00%	93.33%	79.25%	89.78%
	**Thickened**	77.97%	92.00%	74.00%	84.40%	86.84%	75.68%	93.33%	70.00%	83.58%	90.33%
	**Average**	84.11%	83.00%	83.00%	82.86%	87.17%	83.49%	81.67%	81.67%	81.41%	88.42%
**Siamese ResNext & Age**	**Normal**	88.37%	76.00%	90.00%	81.72%	86.88%	80.77%	70.00%	83.33%	75.00%	81.00%
	**Thickened**	78.95%	90.00%	76.00%	84.11%	87.04%	73.53%	83.33%	70.00%	78.12%	80.33%
	**Average**	83.66%	83.00%	83.00%	82.92%	87.00%	77.15%	76.67%	76.67%	76.56%	80.39%

the validation set includes 50 normal patients and 50 patients with thickening, while the test set comprises 30 normal patients and 30 patients with thickening.

#### 3.2.1 Comparison via common indicators

**Figure 5** shows that the Siamese ResNeXt network was identified as the most efficient model for robust and accurate performance in the four common indicators. Siamese ResNeXt network exhibited the highest recall rate reaching a value of 88.0% (**Figure 5A** and **Table 3**). Conversely, the ResNet network was the least efficient, with a recall rate of 80.0%. As shown in **Figure 5B** and **Table 3**, the ResNet model exhibited a precision of 80.00% in the validation group and 79.97% in the test group, indicating that its precision was relatively lower than that of other model groups. The precision of the parallel ResNeXt and Siamese ResNeXt models reached 88.20% and 88.00% respectively, which were the best in the validation group. However, in the test group, the precision of the Siamese ResNeXt model at 85.04% was higher than that of the parallel ResNeXt, which was only 78.36%. **Figure 5C** showed that the F1 Score values of the Siamese ResNeXt model were the highest in both the validation and test groups, achieving 88.0% and 85.0%, respectively. At the same time, the standard ResNet model demonstrated the worst performance, with an F1 Score of 79.97% in the validation group and 74.94% in the test group.

#### 3.2.2 Comparison via clinical indicators

It is clear that the recall rates for the test set were marginally lower than those for the validation set overall, shown in **Figure 5D**. However, despite potential limitations in identifying normal CIMT states, most models exhibited superior performance in detecting thickened conditions. In the ResNet model series, the thickened group exhibited a notable enhancement in predictive recall rates in both validation and test groups, with increments of 8.0%-18.0% and 10.0%-16.77% respectively, when compared to the normal group. Within the Resnext model series, except for the Falltern Rensext model where the outcomes were identical in both scenarios within the validation group, the thickened group consistently achieved a higher recall rate than the normal group, ranging from 4.0%-24.0% across various cases. In the test groups of the Resnext series, the thickened group in the multimodal and standard Resnext models demonstrated an increased recall rate by 6.66%-23.0% over the normal group, while the Parallel ResNeXt and Siamese ResNeXt models showed a decrease in recall rate by 3.33% in the thickened group compared to the normal group.

#### 3.2.3 Comparison of different networks

##### 3.2.3.1 Comparison of Different Deep Neural Algorithms

From the perspective of the deep learning algorithm, the performance of the ResNeXt algorithm consistently outshined the ResNet algorithm in **Figure 5** and **Table 3**. Specifically, in the validation set, the ResNeXt algorithm surpassed the ResNet algorithm by 2% in the standard network architecture, 3% in the parallel network configuration, and 3% in the Siamese network setup. Regarding the test set, the ResNeXt algorithm demonstrated an increase of 5% over the ResNet algorithm in the standard configuration. However, in the parallel configuration, the ResNeXt algorithm fell by 3.34% compared to the ResNet algorithm. In the Siamese configuration, the ResNeXt algorithm showed a significant lead of 6.67% over the ResNet algorithm.

Similarly, in both the validation set and the test set, the AUC of most ResNeXt models were higher than that of ResNet models under the same encoder and decoder (**Figures 5E, F**).

##### 3.2.3.2 Comparison of different encoder and decoder models

In both the validation and test groups, the Siamese network configuration demonstrated a consistently superior performance profile, irrespective of whether ResNet or ResNeXt were used for extracting features, in **Figure 5D**. In the validation group, when the ResNet framework was employed, the standard network architecture yielded the lowest recall rate of 80.0%, whereas the Siamese configuration exhibited the highest recall rate, reaching 85.0%. When the ResNeXt framework was applied, the recall rate of the standard architecture marginally increased to 82.0%, but the Siamese architecture still achieved the highest recall rate, at a value of 88.0%. If the ResNet framework acted as the feature extractor in the test group, the standard architecture had the lowest recall rate at 75.0%. Yet, the parallel architecture achieved the highest recall rate at 81.67%. While the ResNeXt framework was used for prediction CMIT, although the recall rate of the standard architecture increased slightly to 78.33%, the Siamese architecture consistently presented the highest recall rate at 85.0%.

##### 3.2.3.3 Comparison of different data modalities

**Figures 5A, B, C** clearly illustrated that, in the validation group, when the factor of age was embedded into the last complete connection layer, the performance of the model called the Parallel ResNeXt & Age or the Siamese ResNeXt & Age decreased by 3% or 5%, compared with the Parallel ResNeXt or the Siamese ResNeXt. However, the results of the test group varied with that of the validation group. The recall of the Parallel ResNeXt & Age model increased by 3.34% over the Parallel ResNeXt model. Yet the Siamese ResNeXt & Age model decreased by 8.5% compared with the Siamese ResNeXt model.

#### 3.2.4 Confusion matrices and ROC curves of the Siamese ResNeXt

**Figure 6A** presents the confusion matrices for the Siamese ResNeXt model within both validation and test groups. In the validation dataset, the model showed impressive accuracy, accurately predicting ‘Normal’ cases with an actual positive rate of 88.0% and achieving the same accuracy for ‘Thickened’ patients. The false positive and false negative rates were 12.0%, indicating a balanced occurrence of Type I and Type II errors. In the test dataset, more precision is needed. The model proficiently identified ‘Normal’ cases with an 86.67% accuracy and ‘Thickened’ patients at 83.33%. Nevertheless, there was a slight uptick in misclassification rates, with ‘Normal’ cases incorrectly labeled as ‘Thickened’ in 13.33% of instances and ‘Thickened’ cases erroneously identified as ‘Normal’ at a rate of 16.67%.

**Figures 6B, C** show the ROC curves of the Siamese ResNeXt model in the validation and test groups, respectively. In both datasets, the Siamese ResNeXt model consistently recorded the highest AUC values, achieving 90.88% in the validation set and 88.92% in the test set. This performance highlighted the model’s exceptional robustness and efficacy in CIMT prediction.

### 3.3 Results of class activation map

**Figure 7:**
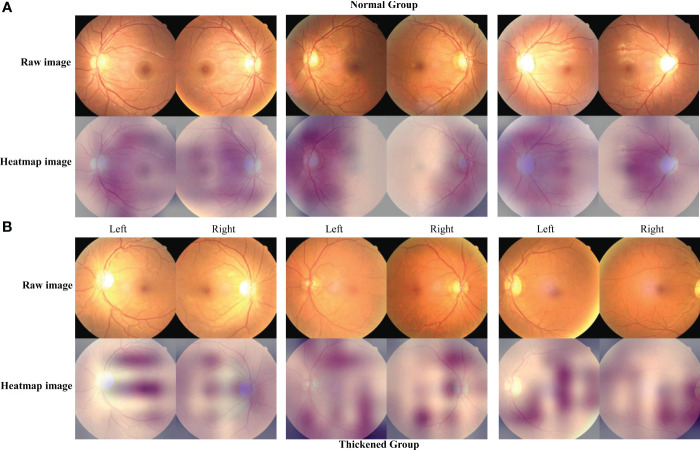
The characteristic heatmap of the Siamese ResNeXt using the Grad-CAM algorithm. **(A, B)** depict the raw images and the heatmaps for the normal and thickened groups, respectively. The heatmaps were the Grad-CAM projections overlaid on these fundus images.

**Figure 7** presents the feature mapping of the Siamese ResNeXt network executed on retinal images. In **Figure 7A**, the Grad-CAM mapping displays the feature distribution for the normal CIMT group. In this group, the features mainly concentrate around the optic disc and vascular areas, exhibiting a centralized and regular pattern on the feature map. This centralization suggests that, in normal CIMT cases, the model focuses more on the optic disc and vascular regions, likely indicative of normal CIMT levels. In contrast, **Figure 7B** shows the Grad-CAM mapping for the thickened CIMT group. The feature map highlights elongated and various point-like circular shapes, with these features being more dispersed across the map. This distinct pattern in feature distribution is attributable to the unique presentation of retinal lesions in the pathological state of CIMT. The observable differences in retinal mapping between the normal and thickened CIMT groups potentially mirror key distinctions in retinal vascular characteristics associated with normal and pathologically altered CIMT states.

## 4 Discussion

### 4.1 Research contributions

We provided a Siamese ResNeXt neural network for predicting CIMT of patients with T2DM from fundus images and confirmed the correlation between fundus microvascular lesions and CIMT.

#### 4.1.1 Clinical significance

It is a well-documented statistic that cardiovascular complications account for the demise of approximately 50% of individuals with T2DM (20) because the continuous chronic hyperglycemia state of patients with diabetes can cause vascular inflammatory responses and endothelial injury (34). CIMT is widely recognized as a precursory biomarker of cardiovascular morbidity, and it is evidenced that incipient alterations in CIMT can be reversed or mitigated through precise pharmacological interventions (35). Therefore, the early detection of CIMT thickening is significant in effectively managing T2DM. Although carotid artery ultrasound is the standard method for CIMT examination, it is not a routine screening for T2DM. Many patients need to attend the early screening of CIMT. A routine and rapid screening method for T2DM is necessary in the clinic.

#### 4.1.2 Biological basis

The ophthalmic arteries, responsible for delivering critical sustenance to ocular components, including the retina and crystalline lens (36), primarily arise from the internal carotid. Carotid stenosis induced by diabetes may enhance the risk of thromboembolic phenomena and attenuated blood flow (37), consequently resulting in ischemic ocular diseases such as retinal artery occlusion and ischemic optic neuropathy (38). Analysis of microvascular changes on fundus images provides valuable information for cardiovascular pathologies (39).

Researchers (40–43) have proved a definite correlation between CIMT and retinal pathologies. Wang (40) demonstrated that the degree of retinal arteriolar hardening has a significant positive correlation with the severity of the carotid atherosclerotic burden, which is characterized by intimal thickening and luminal stenosis (40). The findings of Ichinohasama (41) that individuals with T2DM are suffering from a high risk of CIMT demonstrated the potential of CIMT as an incipient marker for diabetic ocular alterations. Subsequent research elucidated a correlation between the increase in CIMT concomitant and the progression of retinopathy severity among T2DM patients (42, 43). The inverse correlation between CIMT and blood flow and density of retina vascular (44, 45) was further confirmed by Lilla István and Lahme, utilizing sophisticated Optical Coherence Tomography (OCT).

Pathophysiologically and physiologically, predicting CIMT of patients with T2DM from fundus images is underpinned by robust rationale and sufficient evidence.

#### 4.1.3 Intelligent diagnosis technologies

Retinal images are complex and high-dimensional data. If clinicians do not have sufficient clinical experience, they cannot precisely diagnose. Besides, conventional artificial diagnosis methods can not accurately express the complex relationships between the multidimensional data and diseases. The spread of these artificial diagnostic methods may be restricted.

It is acknowledged that Artificial Intelligence (AI) techniques have been pivotal in advancing the diagnostic acuity for various pathologies using retinography, especially in the diagnosis of ocular pathologies and prognostications of holistic health status (12, 13). The study by Wong (46) illuminates the potential of AI-based analyses of the retinal microvasculature to predict cardiovascular disease (CVD) risk factors, direct CVD events, retinal characteristics, and CVD biomarkers, including coronary artery calcium scores (47). Concurrently, Wagner et al’s research, utilizing retinal imaging, has uncovered biomarkers for cardiovascular diseases and dementia, particularly Alzheimer’s disease, thereby underscoring the technology’s capability to reveal systemic diseases. Furthermore, Wu and Liu’s review critically examines the application of deep learning techniques in oculomics derived from retinal images for evaluating systemic health, especially predicting conditions such as sarcopenia. These investigations not only mark significant advancements in retinal image analysis for predictive, preventive, and personalized medicine but also open new avenues for future research and clinical practices (48). They showcase the substantial potential of AI and retinal imaging technologies in refining the accuracy of diagnosing ophthalmological pathologies and in the comprehensive assessment of patients’ overall health conditions.

The investigative collective at West China Hospital, under the aegis of Kang Zhang, has adeptly applied the AneNet architecture for the screening of anemia via retinal vessel Optical Coherence Tomography (OCT) imaging, culminating in an accuracy apex of 98.65% and an exemplary Area Under the Receiver Operating Characteristic Curve (AUC) of 99.83% (49). Meanwhile, this team has pioneered the prognostication of chronic kidney disease through fundoscopic examinations, yielding an AUC span of 0.87 to 0.92 (50), signifying a robust predictive capability.

In this paper, we provided eight models for predicting CIMT, based on ResNet and ResNeXt, using three encoders and decoders, under different data modalities (**Table 2**). Then, the performance of these models was compared. According to the results in Section 3.2.2, Siamese ResNeXt showed the overall best performance. Siamese ResNeXt achieves the highest accuracy, reaching up to 88.0%. The recall of the normal and thickened groups of Siamese ResNeXt is not the highest but can satisfy application requirements. The robustness of Siamese ResNeXt is the best.

Although the performance of the paper is not as high as Diabetic retinopathy detection (an AUC of 99%), which can be attributed to various limiting factors, including the finite dataset size, single-center study design, and the unbalanced distribution of the sample, our accuracy advanced the accuracy previously reported by the consortium at Shenzhen Eye Hospital by an appreciable 14 percentage points (16).

### 4.2 Analysis of different models

#### 4.2.1 Analysis of different network structures

ResNeXt represents an improvement over ResNet, aiming to enhance network representational capacity, computational efficiency, and parameter utilization. They are both classic residual neural networks performing well in image classification tasks. In this paper, ResNet50 and ResNeXt50 were applied in the predicting task CMIT. Overall, ResNeXt50 performed better than ResNet 50. The accuracy of ResNeXt50 is about 2~3% higher than that of ResNet50 in both the validation and test groups because of the ‘cardinality’ (51), which breaks down the width into multiple dimensions. Through group convolution, the network can learn different features more richly.

#### 4.2.2 Analysis of encoding and decoding mode

The input data comprises two images, different from the object recognition task. The dimension of the input data of standard ResNet or ResNeXt is 224×224×3. An appropriate encoder should be designed for this specific task.

Regardless of the deep neural network, the standard encoder performed worst, probably due to the deformation caused by the ‘resize’ operation. Some original features may be stretched, compressed, or distorted when images are deformed.

Overall performance of the Siamese mode is best. The features of a pair of images can be learned simultaneously without deformation. In some tasks, redundant information in the high-dimensional feature space may not significantly contribute to classification tasks. The model can focus more on crucial features despite losing some information by reducing the dimensions.

Parallel Mode performed the best in the recall of the thickened group. Data imbalance between the normal and thickened groups may result in this performance. Meanwhile, overfitting to a specific category is common in Siamese network structures. Because of overfitting, the recall of the thickened group of Parallel ResNeXt is significantly lower than expected.

This study reveals that the Siamese ResNeXt network exhibits superior robustness in terms of both predictive accuracy and model performance. A universal and robust feature was extracted from all samples through the sharing of weight parameters (52), which is of great significance to predict the thickness of the CMIT. Moreover, the attribute of shared weights enables the network to be effectively trained on smaller datasets by reducing the quantity of parameters that need to be learned, which in turn minimizes the risk of overfitting (53). This attribute may contribute to the Siamese ResNeXt network’s heightened accuracy and robustness in predicting CIMT.

#### 4.2.3 Analysis of data modality

Despite a statistically significant age distribution divergence between the CIMT-normal and thickened cohorts in the retrospective analysis, the performance of classification models is reduced if the age factor is embedded in the network, perhaps due to the relationship between age and CIMT prediction is not linear. The age should be processed more appropriately.

### 4.3 Analysis of class activation map

In this investigation, the strategic implementation of Grad-CAM technology on the Siamese ResNeXt network has yielded pivotal insights into the differential feature presentations within fundus imagery, particularly under the diverse physiological states of normal and CIMT. In instances of normal CIMT, the feature mappings prominently coalesce around the vascular environs of the optic disc, suggesting a heightened degree of focalization and structural order. This phenomenon ostensibly mirrors the inherent stability and uniformity of retinal vascular configurations in a salubrious state, implying a preservation of physiological integrity within these vascular zones. Consequently, these regions within the fundus imagery are algorithmically recognized as denotative of a normative vascular state devoid of significant carotid arterial thickening.

Conversely, the feature mappings associated with thickened CIMT conditions are markedly disparate, characterized by dispersion and an absence of regular patterning, potentially signaling underlying pathological shifts. This dispersed mapping paradigm may directly correlate with pathological processes intrinsic to increased CIMT, culminating in the manifestation of irregular and heterogeneous vascular attributes within the fundus images. Such findings indicate potential alterations in the retinal vascular architecture consequent to CIMT augmentation, engendering a diverse array of morphological and structural retinal modifications. These revelations augment our comprehension of the intricate nexus between cardiovascular health and retinal vascular characteristics and significantly enhance the potential utility of fundus images as a sophisticated, non-invasive modality for cardiovascular risk assessment. This advancement holds substantial promise for enriching the armamentarium of clinical diagnostics and refining cardiovascular medicine preventative strategies.

## 5 Conclusions

The predictive analysis of CIMT through fundoscopic imaging bears critical implications for the preemptive risk stratification of macrovascular complications among patients diagnosed with T2DM. In this research, a range of deep neural network structures were applied to forecast the thickening of CIMT in T2DM patients. The architectures included conventional neural networks, neural networks with parallel structures, siamese neural networks, and multimodal neural networks integrating age factors. The siamese ResNeXt model, in particular, showed exceptional efficacy in predicting CIMT thickening, recording a recall rate of 88.0% and an AUC of 90.88% on the validation set and exhibited notable robustness in the testing phase. Nevertheless, with the impetus for future research to focus on enhancing interpretable machine learning features, alongside the enlargement of sample cohorts and multi-center study inclusion, significant advancements in the precision of CIMT predictive models based on fundoscopic imaging are expected. This research delineates a foundational framework for the integration of ocular fundoscopic assessments in the realm of cardiovascular diagnostics. It suggests expansive prospects for its application in clinical settings, enriching the early cardiovascular disease intervention methodologies.

## Data availability statement

The raw data supporting the conclusions of this article will be made available by the authors, without undue reservation.

## Ethics statement

The studies involving humans were approved by the Ethics Committee of the Second Affiliated Hospital of Anhui Medical University. The studies were conducted in accordance with the local legislation and institutional requirements. The ethics committee/institutional review board waived the requirement of written informed consent for participation from the participants or the participants’ legal guardians/next of kin because The Ethics Committee of the Second Affiliated Hospital of Anhui Medical University has granted an exemption from written informed consent for this study based on the following grounds: (1) The study is retrospective, precluding real-time contact with participants by the research team; (2) The scope of the research is confined to the utilization of medical records of hospitalized patients, encompassing details such as age, gender, carotid ultrasound, and fundus imagery data, all of which have been anonymized to maintain privacy and security; (3) During their hospitalization, patients had already provided a comprehensive consent, authorizing their clinical data for research purposes.

## Author contributions

AG: Investigation, Data curation, Funding acquisition, Methodology, Software, Validation, Writing – original draft, Writing – review & editing. WF: Data curation, Writing – original draft, Methodology, Validation, Formal analysis. HL: Methodology, Writing – review & editing. NG: Methodology, Project administration, Resources, Supervision, Visualization, Writing – review & editing, Formal analysis. TP: Data curation, Methodology, Project administration, Resources, Supervision, Writing – review & editing.

## Supplementary material

The Supplementary Material for this article can be found online at: https://www.frontiersin.org/articles/10.3389/fendo.2024.1364519/full#supplementary-material. For the code used in this study, please refer to the following GitHub repository: https://github.com/gongajuan/cimt-predict.
